# Reviewers

**DOI:** 10.1177/2041669517747171

**Published:** 2017-12-26

Perception and i-Perception would like to thank the following for reviewing in 2017:

Adolph, Dirk

Ainsworth, Kirsty

Al Ain, Syrina

Alberti, Concetta

Alder, Dave

Allik, Jyri

Allison, Robert

Andersson, Linus

Anobile, Giovanni

Ansorge, Ulrich

Anstis, Stuart

Apthorp, Deborah

Arrighi, Roberto

Ashida, Hiroshi

Ashmead, Daniel

Aston, Stacey

Atkinson, Tony

Attwood, Angela

Axelrod, Vadim

Bach, Dominik

Bach, Michael

Bachmann, Talis

Backus, Benjamin

Badcock, David

Baker, Christopher

Baker, Daniel

Balas, Benjamin

Bankieris, Katie

Banks, Martin

Barla, Pascal

Barraclough, Nick

Barratt, Daniel

Bateson, Melissa

Baudouin, Jean-Yves

Bauer, Clemens C. C.

Bayliss, Andrew

Bays, Paul

Bear, Gordon

Beattie, Louise

Beck, Brianna

Bente, Gary

Berger, Jacob

Bertamini, Marco

Berti, Stefan

Beurskens, Carien

Bex, Peter

Bhattacharya, Joydeep

Billington, Jac

Binda, Paola

Bindemann, Markus

Bingham, Geoffrey

Blank, Helen

Blasi, Damian

Block, Richard

Boesveldt, Sanne

Bonato, Frederick

Boothroyd, Lynda

Boyaci, Huseyin

Braddick, Oliver

Bramao, Ines

Brandt, Stephan

Brang, David

Brascamp, Jan

Bravo, Mary

Brenner, Eli

Bret, Amélie

Bronstad, Matthew

Brooks, Joseph

Brooks, Kevin

Brouwer, Anne-Marie

Brown, Angela

Bruno, Nicola

Bubl, Emanuel

Bukowski, Henryk

Burleigh, Tyler

Caclin, Anne

Cai, Mingbo

Calvillo, Dustin

Caplovitz, Gideon

Carbon, Claus-Christian

Cardello, Armand

Carré, Justin

Casati, Roberto

Castellanos, Irina

Castelo, Paula

Cattaneo, Zaira

Cavanagh, Patrick

Caziot, Baptiste

Chamberlain, Rebecca

Chen, Chien-Chung

Chen, Kewei

Chen, Zhongting

Chetverikov, Andrey

Cho, Yang Seok

Ciaramitaro, Vivian

Clark, Andrew P.

Colloff, Melissa

Cometto-Muniz, Enrique

Conway, Bevil

Cook, Richard

Cormack, Lawrence

Costa, Manuela

Crookes, Kate

Croy, Ilona

Curran, William

Cuthill, Innes

Cutting, James

Cuturi, Luigi

Damon, Fabrice

Dan, Alex

Danilova, Marina

Davis, Helen

Davis, Josh

de Brouwer, Anouk

de Gelder, Beatrice

de Gutis, Joe

De la Rosa, Stephan

deFockert, Jan

Delannoy, Jaime

Delgado, Rafael

Delhaye, Benoit

Deroy, Ophelia

Dickinson, Christopher

Dillenburger, Barbara

Dils, Alexia

Dingemanse, Mark

Dresp-Langley, Birgitta

Drewing, Knut

Dudschig, Carolin

Dugué, Laura

Duke, Phillip

Dupoux, Emmanuel

Durant, Szonya

Durgin, Frank

Dzhelyova, Milena

Edmonds, Andrew

Einhäuser, Wolfgang

Ekroll, Vebjørn

Ekstrand, Chelsea

Elliot, Andrew

Emery, Kara

Endres, Dominik

Engbert, Ralf

Ennis, Robert

Enns, James

Erlikhman, Gennady

Estudillo, Alejandro

Ewing, Louise

Fadde, Peter

Fahrenfort, Johannes

Falkenberg, Helle

Farell, Bart

Farmer, Harry

Fassbender, Ina

Feldman, Anatole

Feldmann-Wuestenfeld, Tobias

Felnhofer, Anna

Fias, Wim

Finlayson, Nonie

Firestone, Chaz

Fiset, Daniel

Fisher, Katie

Fleming, Roland

Forde, Ciaran

Foulkes, Paul

Franceschini, Sandro

Freeman, Tom

Freiherr, Jessica

Frowd, Charlie

Froyen, Vicky

Funk, Friederike

Furlan, Michele

Furley, Philip

Fusco, Gabriele

Gagnier, Kristin

Galit, Yovel

Gallagher, Reagan

Galmonte, Alessandra

Gao, Tao

Garcia-Perez, Miguel

Gentaz, Edouard

Georgescu, Alexandra

Georgeson, Mark

Gerbino, Walter

Gerger, Gernot

Geuss, Michael

Giacco, Grazia

Gilaie-Dotan, Sharon

Gilchrist, Alan

Gill, Daniel

Gilmore, Rick

Giuliani, Nicole

Glennerster, Andrew

Godfroy, Martine

Goodale, Melvyn

Gori, Monica

Gori, Simone

Gosselin, Frederic

Goutcher, Ross

Grabowecky, Marcia

Graf, Erich

Grassi, Massimo

Gray, Katie

Griffin, Lewis

Griffiths, Tom

Grove, Philip

Gundlach, Christopher

Gur, Moshe

Haigh, Sarah

Hamburger, Kai

Hammett, Stephen

Hancock, Peter

Harrison, William

Harsanyi, Geza

Hartmann, Matthias

Hayes, John

Hayn-Leichsenring, Gregor

Hayward, Dana A.

Hayward, Vincent

Hayward, William

He, Zijiang

Heard, Priscilla

Heaton, Pam

Hecht, Heiko

Heller, Morton

Herbort, Oliver

Herbst, Sophie

Heron, James

Heuer, Herbert

Hibbard, Paul

Hidaka, Souta

Hill, Harold

Holcombe, Alex

Holle, Henning

Hollins, Mark

Holmes, Nicholas

Hommel, Bernhard

Hooge, Ignace

Hoogenhout, Michelle

Horry, Ruth

Hossner, Ernst

Hostetter, Autumn

Houpt, Joseph

Hout, Michael

Howard, Christina

Hsieh, Po-Jang

Hubbard, Timothy

Huebner, Ronald

Hughes, Darin E.

Hulleman, Johan

Hummel, Thomas

Hunt, Amelia

Hurlbert, Anya

Huys, Raoul

Hwang, Alex

Ito, Hiroyuki

Jackson, Philip

Jacobs, Richard

Jakesch, Martina

Jax, Steve

Jenkins, Rob

Jessen, Sarah

Johnson, Aaron

Johnston, Alan

Jones, Alex

Jones, Benedict

Jones, Matthew

Kaneko, Sae

Kargas, Nico

Kaschak, Michael

Kashima, Hideaki

Kawabe, Takahiro

Keenan, Julian

Keil, Matthias

Kennedy, John

Kerzel, Dirk

Keshner, Emily

Khuu, Sieu

Kim, Juno

Kingdom, Fred

Kirkby, Julie

Kitaoka, Akiyoshi

Kitazaki, Michiteru

Klein, Raymond

Knoblauch, Kenneth

Koerner, Christof

Kogo, Naoki

Kopiske, Karl

Kramer, Robin

Kreitz, Carina

Krekelberg, Bart

Kret, Mariska

Kristjansson, Arni

Kuai, Shuguang

Kuhlen, Anna

Kuriki, Ichiro

La Buissonnière-Ariza, Valerie

Lages, Martin

Lamm, Claus

Landwehr, Klaus

Lappe, Markus

Lappin, Joseph

Layton, Oliver

Lewis, Elizabeth

Lewis, Gary

Lewis, Michael

Li, Hsin-Hung

Li, Zhi

Linares, Daniel

Lindell, Annukka

Lindsey, Delwin

Linkenauger, Sally

Lipp, Ottmar

Little, Anthony

Liu, Ye

Liuzza, Marco Tullio

Lloyd, Donna

Locher, Paul

Locke, John

Loffing, Florian

Logvinenko, Alexander

Longmore, Chris

Loveland, Katherine

Lovell, George

Lübke, Katrin

Lucchina, Laurie

Luke, Steven

Lundström, Johan

Lunghi, Claudia

Lupiáñez, Juan

MacDonald, Lindsay

MacDorman, Karl

Maertens, Marianne

Magliano, Joe

Maguinness, Corrina

Maiello, Guido

Majid, Asifa

Makin, Alexis

Mareschal, Isabelle

Markovic, Slobodan

Marks, Lawrence

Marlow, Phillip

Martin, Douglas

Martinez-Conde, Susana

Masuda, Yuji

Mather, George

Mathes, Birgit

Mathôt, Sebastiaan

Matsumiya, Kazumichi

Matsuyoshi, Daisuke

Matthews, Nestor

McAnany, Jason

McClelland, Tom

McGraw, Paul

McManus, Chris

Meese, Tim

Melcher, David

Menetrier, Emmanuelle

Mikellidou, Kyriaki

Mills, Mark

Mitchell, Teresa Vann

Miyamoto, Yuri

Mollon, John

Mondloch, Catherine

Moors, Pieter

Morgan, Michael

Morikawa, Kazunori

Morimoto, Takuma

Mottron, Laurent

Moulson, Margaret

Mudrik, Liad

Mulatti, Claudio

Munar, Enric

Munger, Margaret

Murray, Richard

Muth, Claudia

Mutic, Smiljana

Naber, Marnix

Nadal, Marcos

Nakamura, Shinji

Nascimento, Sergio

Nava, Elena

Naveteur, Janick

Neta, Maital

Newell, Fiona

Ni, Rui

Niehorster, Diederick C.

Nöel, Jean-Paul

Nordin, Steven

Ntonia, Iro

Nyström, Marcus

O’Herron, Philip

Oberfeld-Twistel, Daniel

Occelli, Valeria

O'Dea, John

Okajima, Katsunori

Okazawa, Gouki

Olkkonen, Maria

Olofsson, Jonas

Olsson, Mats

Or, Charles

Osorio, Daniel

Ossandon, Jose

O'Sullivan, Noreen

Paffen, Chris

Palmisano, Stephen

Papathomas, Thomas

Paramei, Galina

Park, Justin

Parma, Valentina

Parraga, Alejandro

Pascalis, Olivier

Pavani, Francesco

Pearson, Joel

Pecher, Diane

Peelen, Marius

Peirce, Jonathan

Pelowski, Matthew

Peltola, Mikko

Penna, Maria Pietronilla

Pepperell, Robert

Pesenti, Mauro

Philbeck, John

Piech, Richard

Pinna, Baingio

Pinto, Jayant

Pollick, Frank

Potter, Mary

Preston, Catherine

Prince, Jon

Prins, Nicolaas

Raab, Markus

Radonjić, Ana

Radvansky, Gabriel

Read, Jenny

Redfern, Annabelle

Redies, Christoph

Reed, Danielle

Reeves, Adam

Regier, Terry

Rider, Andy

Ritchie, Kay

Rizzi, Alessandro

Robinson, Chris

Rogers, Brian

Roncato, Sergio

Roseboom, Warrick

Rosenbaum, David

Rossetti, Yves

Rotella, Amanda

Rothen, Nicolas

Royden, Constance (Stanzi)

Rudd, Michael

Rushton, Simon

Russell, Richard

Salomon, Roy

Samson, Fabienne

Sanders, Jet

Scarfe, Peter

Schawelka, Karl

Schloss, Karen

Schneider, Jennifer

Schneider, Keith

Schofield, Andrew

Schultz, Johannes

Schutz, Michael

Schwark, Jeremy

Schwarzkopf, Dietrich

Sedgwick, Hal

Seibold, Verena

Self, Matthew

Sellaro, Roberta

Seo, Han-Seok

Serrano-Pedraza, Ignacio

Servos, Philip

Seubert, Janina

Shioiri, Satoshi

Shore, David

Short, Lindsey

Silvia, Paul

Simmons, David

Sinha, Pawan

Skinner, Andy

Skylark, William

Smeets, Monique

Smith, Dan

So, Richard

Solomon, Josh

Song, Joo-Hyun

Speed, Laura

Spence, Charles

Spering, Miriam

Srinivasan, Narayanan

Steadman, Philip

Stephen, Ian

Stins, John

Stothart, George

Sun, Hongjin

Sunram-Lea, Sandra

Suslow, Thomas

Swami, Viren

Swapp, David

Sweeny, Timothy

Takahashi, Kohske

Takeuchi, Tatsuto

Tan, Hong

Tassinary, Louis

Tatler, Benjamin

Taubert, Jessica

Ten Brink, Teuni

Thompson, Peter

Thornton, Ian

Todorović, Dejan

Toma, Simone

Tordoff, Michael

Toscani, Matteo

Towler, John

Townsend, James

Tredoux, Colin

Tremoulet, Patrice

Tse, Peter

Tsougras, Costas

Tsujimura, Sei-Ichi

Tyler, Christopher

Tyler, Michael

Unger, Marianne

Unkelbach, Christian

Utochkin, Igor

Uuskula, Mari

van den Berg, Ronald

Van den stock, Jan

Van der Burg, Erik

Van der Helm, Peter

van der Kooij, Katinka

van der Linden, Sander

Van der Stoep, Nathan

van Doorn, Andrea

van Ee, Raymond

van Leeuwen, Cees

Van Lier, Rob

van Wassenhove, Virginie

Vangorp, Peter

Varakin, D Alexander

Vatakis, Argiro

Vecchi, Tomaso

Vergeer, Mark

Verlage, Sarah

Verstraten, Frans

Vierck, Charles

Vigliocco, Gabriella

Vishwanath, Dhanraj

Vocks, Silja

Volberg, Gregor

Volcic, Robert

Volkova, Ekaterina

von Mühlenen, Adrian

Voss, Patrice

Vuoskoski, Jonna

Wachtler, Thomas

Wade, Nicholas

Wagemans, Johan

Wagman, Jeffrey

Wagner, Mark

Wagner, Valentin

Walker, Peter

Walther, Dirk

Walther, Sebastian

Ward, Jamie

Wardle, Susan

Warren, Paul

Watt, Roger

Webster, Michael

Werner, John

White, Alex

White, David

Whitwell, Robert

Wiecek, Emily

Wiese, Holger

Wijntjes, Maarten

Wilkins, Arnold

Wilson, Hugh

Wise, Paul

Witzel, Christoph

Wolfe, Jeremy

Wright, Geoffrey

Wuerger, Sophie

Xiao, Bei

Yamada, Yuki

Yau, Jeffrey

Yu, Cong

Zavagno, Daniele

Zetzsche, Christoph

Zhang, Xilin

Zhao, Shuo

Zopf, Regine

